# Understanding Pharmaco-Epigenomic Response of Antipsychotic Drugs Using Genome-Wide MicroRNA Expression Profile in Liver Cell Line

**DOI:** 10.3389/fnmol.2022.786632

**Published:** 2022-03-22

**Authors:** Babu Swathy, Moinak Banerjee

**Affiliations:** Neurobiology and Genetics Division, Rajiv Gandhi Center for Biotechnology, Thiruvananthapuram, India

**Keywords:** antipsychotics, schizophrenia, microRNA, pharmacoepigenomics, haloperidol, clozapine, epigenetics, drug metabolism

## Abstract

Interindividual variability in drug response is a major concern among patients undergoing antipsychotic drug treatment. Apart from genetic and physiological factors, this variability in drug response could also be attributed to epigenetic mechanisms. The microRNAs (miRNAs) are key epigenetic markers that play an important role in pathogenesis and drug response. Several studies have shown that miRNAs are implicated in regulating the expression of various genes involved in drug metabolism and transport. In a conventional clinical setup, it is extremely difficult to distinguish the role of miRNA in pathogenesis and drug response as it is difficult to obtain drug naïve patients. To resolve this issue, we aimed to identify the role of antipsychotic drug treatment in inducing miRNA expression under an *in vitro* condition using a hepatic cell line. A liver cell line was treated with a maximum tolerable drug dosage model for haloperidol, clozapine in monotherapy, and their combination in polytherapy. Genome-wide miRNA profiling was performed using 60,000 miRNA probes in the microarray format in different treatment groups. Several miRNAs were observed to be differentially expressed impacting the pharmacokinetic, pharmacodynamics, and epigenomics properties of antipsychotic drug treatment. Interestingly, some of these miRNA expression patterns were similar to reported miRNA observations on schizophrenia pathogenesis. This study unravels the potential role of miRNAs in the mechanism of action of the antipsychotic drug and could also reflect in drug-induced side effects. This study also signifies the importance of pharmacoepigenomics approach while evaluating the role of miRNAs in pathogenesis.

## Introduction

Antipsychotic drugs are considered as the first-line treatment for various psychotic disorders including schizophrenia. Patients receiving antipsychotic drugs show heterogeneity in drug response which could be due to genetic or non-genetic factors influencing drug response. Pharmacogenetic studies have identified the potential role of polymorphic genes in treatment response and drug-induced adverse events in patients diagnosed with schizophrenia (Malhotra et al., 2004). But, these observations provide limited success in monitoring side effects, which possibly suggests that there could also be an alternate mechanism to find variability in therapeutic response. Epigenetic mechanisms can offer an alternate mechanism for interindividual drug variability, which cannot be explained by genetic polymorphisms (Ivanov et al., 2012). The altered drug response could be due to the inherent epigenome of the host or the drug-induced epigenetic changes namely pharmacoepigenetics changes (Banerjee, 2019; Swathy et al., 2019). The main epigenetic mechanisms include DNA methylation, histone modifications, and microRNA (miRNA) expression.

Several studies have reported alterations in miRNA expressions in schizophrenia, where the patients were selected from conventional treatment background (Caputo et al., 2015; Swathy and Banerjee, 2017b). The majority of these studies were on postmortem brain tissues or peripheral blood. In a conventional clinical setup, the patient might be on multiple medications, and therefore, it is difficult to evaluate the effect of individual antipsychotics and their role in modulating the expression profile of various miRNAs and their target genes. Given the universal roles of miRNAs in diseased conditions and therapeutic response, it is important to dissect and distinguish these two features very precisely. There are very less studies yet on how miRNAs affect patient outcomes following antipsychotic drug treatments (Sun et al., 2015). The subject of miRNA pharmacoepigenomics combines the knowledge on miRNA-mediated gene regulation and drug response (Zhang and Dolan, 2010). Experimental evidence on the roles of miRNAs in regulating pharmacology-related genes and drug response is emerging (Ivanov et al., 2012; He et al., 2015; Swathy et al., 2017). To investigate this further at the epigenome-wide miRNA level, this study was undertaken to globally profile the miRNAs that can be influenced by antipsychotic drugs, impacting the first-pass effect on metabolism, using a liver cell line. Many of the antipsychotic drugs are known to interfere with metabolic functions and can induce severe metabolic side effects. This study might help in distinguishing and differentiating the role of miRNA in drug response and pathogenesis and also help in better understanding the metabolic side effects.

## Materials and Methods

### Cell Culturing and Antipsychotic Drug Treatment

The human hepatocellular carcinoma cell line, HepG2, a human liver cell line was chosen as the experimental cell line as most antipsychotic drugs undergo first-pass metabolism in the liver. HepG2 [American Type Culture Collection (ATCC), CRL-1573] was maintained in Dulbecco’s modification of Eagle’s medium (DMEM; Gibco) supplemented with 10% fetal bovine serum (Gibco) and 1X antibiotic antimycotic solution (Invitrogen) in a 37°C humidified incubator with 5% CO_2_. Short tandem repeat (STR) DNA profiling, according to American Tissue Culture Collection (ATCC) standards, was performed to confirm the identity of the cell line. HepG2 cells were treated with haloperidol and clozapine in monotherapy and polytherapy for 24 h. For monotherapy, 25 μM haloperidol (HLP) and 25 μM clozapine (CLZ) were used, and for combinatorial drug treatment, 25 μM HLP + 25 μM CLZ were used. HepG2 was treated for monotherapy and polytherapy at 25 μM concentration for 24 h. miRNA microarray was performed to identify the differentially expressed miRNAs under each drug treatment condition. The potential biological pathways associated with each drug treatment condition were identified. The choice of drug concentration was based on the effective dose determined from our previous studies (Swathy et al., 2017). For the control experiment, a fresh medium (including DMEM as control) was added.

### RNA Isolation

Total RNA extraction was performed from cell lines (treated or untreated) using the TRIzol reagent (Invitrogen), according to the manufacturer’s instructions. RNase-free DNase I (QIAGEN, Germany) was added to remove the genomic or cell-free DNA contamination. The integrity and quality of RNA were evaluated using RNA 6000 Nano Lab Chip on the 2100 Bioanalyzer (Agilent, Palo Alto) following the manufacturer’s protocol. Total RNA purity was assessed at absorbance (A) at 260 and 280 nm, respectively, using the NanoDrop^®^ ND-2000 UV-Vis spectrophotometer (NanoDrop Technologies). RNA was considered to be of good quality based on the rRNA 28S/18S ratios and RNA integrity number (RIN). The threshold for RNA quality suitable for microarray analysis was considered to be a RIN ≥ 7.0 indicating moderate to good RNA quality.

### Labeling and Microarray Hybridization

MicroRNA profiling was performed using the Agilent Technologies miRNA profiling system (Genotypic Technology Private Limited, Bangalore). The miRNA labeling was carried out using the miRNA Complete Labeling and Hyb Kit (Agilent Technologies, Cat. No. 5190-0456). The total RNA sample was diluted to 100 ng/μl in nuclease-free water. Approximately, 200 ng of total RNA was treated using the calf intestinal alkaline phosphatase (CIP) master mix (Agilent Technologies, Cat. No. 5190-0456) by incubating at 37°C for 30 min. The dephosphorylated miRNA samples were denatured by adding Dimethylsulfoxide (DMSO) and heating at 100°C for 10 min and transferred to an ice-water bath. The Ligation Master Mix (Agilent Technologies, Cat. No. 5190-0456) containing cyanine 3-pCp was added to the denatured miRNA samples and incubated at 16°C for 2 h. The cyanine 3-pCp labeled miRNA samples were dried completely in the vacuum concentrator (Eppendorf, Concentrator Plus, Cat. No. 5305000) at 45°C for 2 h. The dried samples were resuspended in nuclease free water (NFW) and mixed with hybridization mix containing blocking solution (Agilent Technologies, Cat. No. 5190-0456) and Hi-RPM hybridization buffer (Agilent Technologies, Cat. No. 5190-0403) and incubated at 100°C for 5 min followed by snap chill on ice for 5 min. The samples were hybridized on the human miRNA 8 × 60k arrays. The hybridization was carried out at 55°C for 20 h. After hybridization, the slides were washed using Gene 2 Expression Wash Buffer 1 (Agilent Technologies, Cat. No. 5188-5325) at room temperature for 5 min and Gene Expression Wash Buffer 2 (Agilent Technologies, Cat. No. 5188-5326) at 37°C for 5 min. The slides were then washed with acetonitrile for 30 s. The microarray slide was scanned using the Agilent Scanner (Agilent Technologies, Cat. No. G2565CA).

### Microarray Data Analysis

Data extraction from images was performed using Agilent Feature Extraction software. Feature extracted data were analyzed using Agilent GeneSpring GX version software. Normalization of the data was performed using GeneSpring GX using the 90th percentile shift and normalization to specific samples. [Percentile shift normalization is a global normalization, where the locations of all the spot intensities in an array are adjusted. This normalization takes each column in an experiment independently and computes the *n*th percentile of the expression values for this array, across all spots (where *n* has a range from 0–100, and *n* = 90 is the median). It subtracts this value from the expression value of each entity]. Significant differentially expressed miRNA upregulated and downregulated within the group of samples were identified. [Up fold > 2 (log base2) and Down < −1 (log base2)]. Differentially regulated miRNAs were clustered using hierarchical clustering based on the Pearson coefficient correlation algorithm to identify significant gene expression patterns. Data comparing differences in levels of expression of miRNAs between control and drug-treated samples were analyzed using paired Student’s *t*-test using Genespring version GX 11 software (Agilent Inc.). A volcano plot was plotted showing individual miRNA expression with significance and fold change using GraphPad Prism 9. Venn diagrams were created using Venny 2.1.0^1^.

### Information Resources and Databases for Pathway Analysis

The gene targets for the differentially expressed miRNAs were identified using Genespring GX, and these genes were subjected for pathway analysis using DAVID database version 6.7^2^. Information on validated miRNA-target interactions on Kyoto encyclopedia of genes and genomes (KEGG) pathways including ATP-binding cassette transporters (ABC transporters), drug metabolism cytochrome P450, drug metabolism other enzymes, and metabolic pathways, as well as epigenetic machinery belonging to WIKI pathway, were retrieved from gene-miRNA pathway predicted target module available in miRwalk database^3^. Thus, the miRNAs obtained were compared with the miRNAs altered in drug treatment condition using Microsoft excel tools, and miRNAs belonging to each pathway was identified.

## Results

### Identification of Differentially Expressed miRNAs

**FIGURE 1:**
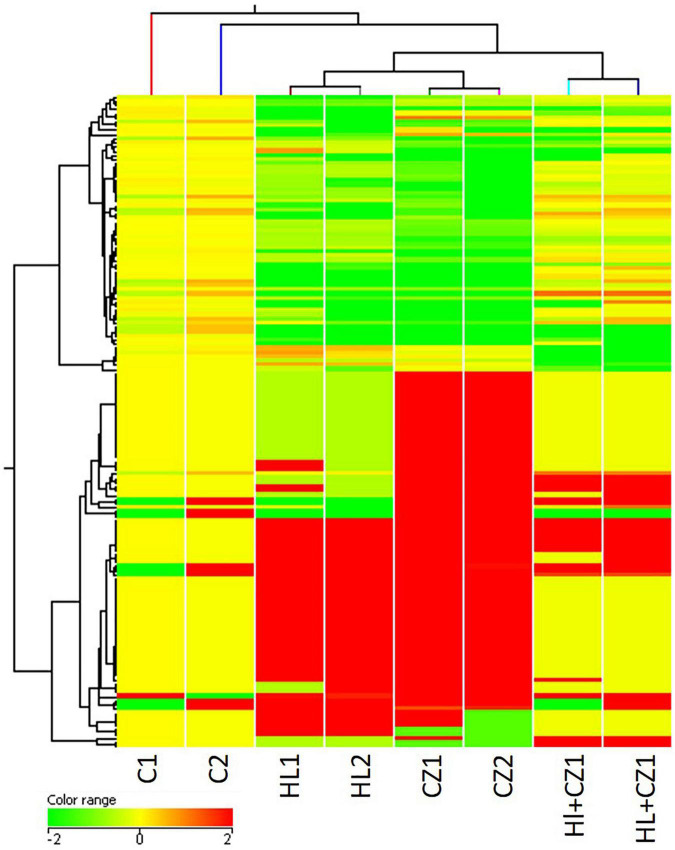
Heat map showing altered microRNAs (miRNAs) in the different treatment groups (HL – haloperidol, CZ – clozapine, and HL + CZ) and control (C). The color range scale depicts the expression level of a miRNA across all samples. Red color represents expression above the mean, and green color represents expression lower than mean. Significant changes in expression levels from high to low and vice-versa can be seen as distinct blocks across samples for different miRNAs.

**FIGURE 2:**
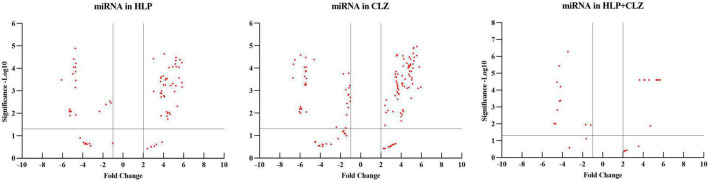
Volcano plot of differentially expressed miRNAs beyond the threshold in the different treatment groups (HLP – haloperidol, CLZ – clozapine, and HLP + CLZ) and control (C).

MicroRNA expression profiling identified several miRNAs altered with each drug treatment compared with control. The list of significantly altered miRNAs (fold change ≥ + 2 and −1, and *P*-values < 0.05) under each drug treatment condition are given in Supplementary Table S1. The total number of miRNAs upregulated and downregulated with each treatment is shown in Table 1. A total of 42, 70, and 15 miRNAs were upregulated with 25 μM HLP, 25 μM CLZ, and 25 μM HLP + 25 μM CLZ, respectively. In contrast, 20, 36, and 11 miRNAs were downregulated with 25 μM HLP, 25 μM CLZ, and 25 μM HLP + 25 μM CLZ, respectively. Eight miRNAs were commonly upregulated, and three were commonly downregulated in all conditions. This differential miRNA expression profile with pairwise comparison is displayed in a heat map along with its clustering features in Figure 1. The color range scale depicts the expression level of a miRNA across all samples. Red color represents expression above the mean, and green color represents expression lower than mean. Significant changes in expression levels from high to low and vice-versa can be seen as distinct blocks across samples for different miRNAs. These differentially expressed miRNAs beyond the threshold of fold change are further depicted in a volcano plot (Figure 2).

**TABLE 1:** Number of microRNAs (miRNAs) upregulated and downregulated in each antipsychotic drug treatment group.

Analysis Plan	Up regulated	Down regulated
25 μM HLP Vs. Control	42	20
25 μM CLZ Vs. Control	70	36
25 μM HLP + 25μM CLZ Vs. Control	15	11

**FIGURE 3:**
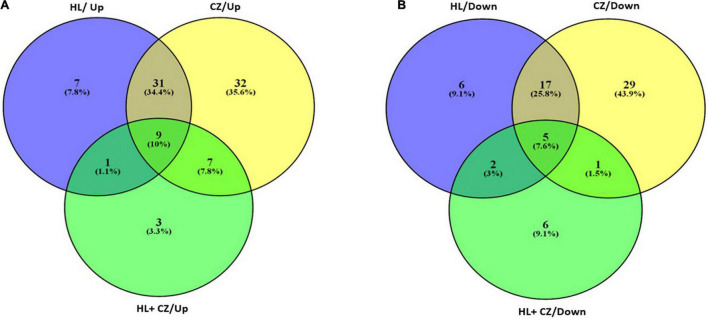
Venn diagram representing the number of miRNAs upregulated **(A)** and downregulated **(B)** in each drug treatment condition.

All the miRNA expressions that are significantly altered in different treatment groups are further represented in a Venn diagram showing overlapping miRNA that is expressed across all treatment groups, or in two treatment groups, or exclusively in individual treatment groups (Figure 3). The list of commonly upregulated and downregulated miRNAs and their fold change and *P*-value are presented in Table 2. The miRNAs that are exclusively altered in each treatment group depicting drug-specific miRNA signatures are shown in Table 3. This includes a total of 6, 30, and 3 miRNAs that were exclusively upregulated and 6, 23, and 5 miRNAs that were exclusively downregulated in 25 μM HLP, 25 μM CLZ, and 25 μM HLP + 25 μM CLZ, respectively (Table 3).

**TABLE 2:** miRNAs that are commonly but significantly upregulated and downregulated in all antipsychotic drug treatment groups.

miRNAs	25 μM HLP Vs. control		25 μM CLZ Vs. control		25 μM HLP + CLZ Vs. control	
	Fold change	*P* value	Fold change	*P* value	Fold change	*P* value
**Commonly up regulated miRNAs**						
hsa-miR-7152-3p	5.55	0.0003	5.56	0.0000	5.44	0.0000
hsa-miR-6794-5p	5.23	0.0006	5.17	0.0001	5.66	0.0000
hsa-miR-6807-5p	5.83	0.0001	5.79	0.0001	5.68	0.0000
hsa-miR-5088-5p	5.83	0.0004	5.68	0.0008	5.41	0.0001
hsa-miR-1288-3p	5.62	0.0000	5.24	0.0000	5.30	0.0002
hsa-miR-4419a	5.22	0.0000	4.74	0.0001	4.10	0.0002
hsa-miR-3141	4.43	0.0182	5.05	0.0003	4.17	0.0003
hsa-miR-3156-5p	5.85	0.0007	5.30	0.0000	5.59	0.0004
**Commonly down regulated miRNAs**						
hsa-miR-7974	−4.67	0.0003	−5.37	0.0003	−4.20	0.0004
hsa-miR-6848-3p	−5.29	0.0079	−5.98	0.0062	−4.81	0.0095
hsa-miR-4758-3p	−5.15	0.0084	−5.84	0.0065	−4.67	0.0101

**TABLE 3:** List of miRNAs exclusively but significantly altered in each antipsychotic drug treatment group.

miRNAs	Fold change	*P* value	miRNAs	Fold change	*P* value
**Exclusively up regulated in 25 μM HLP Vs. control**			**Exclusively down regulated in 25 μM HLP Vs. control**		
hsa-miR-200a-5p	3.77	0.0128	hsa-let-7b-3p	−2.34	0.0083
hsa-miR-335-5p	3.69	0.0013	hsa-miR-4787-5p	−1.31	0.0029
hsa-miR-374c-5p	3.98	0.0002	hsa-miR-6068	−1.19	0.0035
hsa-miR-452-5p	4.39	0.0099	hsa-miR-6752-3p	−4.87	0.0000
hsa-miR-4800-5p	4.30	0.0085	hsa-miR-6789-5p	−4.96	0.0002
hsa-miR-619-5p	3.02	0.0000	hsa-miR-1229-5p	−3.75	0.0157
**Exclusively up regulated in 25 μM CLZ Vs. control**					
hsa-let-7d-3p	2.52	0.0094	hsa-miR-4327	4.83	0.0001
hsa-miR-1224-5p	4.77	0.0001	hsa-miR-4462	4.06	0.0004
hsa-miR-1236-5p	4.56	5.95E-05	hsa-miR-4481	3.41	0.0001
hsa-miR-134-5p	4.98	0.0001	hsa-miR-4499	4.79	3.21E-05
hsa-miR-135a-3p	2.41	0.0349	hsa-miR-4651	2.40	0.0045
hsa-miR-1469	3.52	0.0002	hsa-miR-5195-3p	3.98	0.0141
hsa-miR-183-3p	2.69	0.0075	hsa-miR-550a-3-5p	3.48	0.0001
hsa-miR-202-3p	4.01	0.0222	hsa-miR-6076	5.17	2.21E-05
hsa-miR-2392	4.14	0.0004	hsa-miR-6741-5p	3.41	0.001664
hsa-miR-328-5p	3.65	0.0001	hsa-miR-6768-5p	4.21	8.09E-05
hsa-miR-33a-5p	4.59	0.0007	hsa-miR-6775-5p	3.77	0.001448
hsa-miR-3648	4.23	0.0084	hsa-miR-6786-5p	3.94	0.00989
hsa-miR-378b	3.63	0.0024	hsa-miR-6801-3p	3.58	0.001109
hsa-miR-4257	4.17	2.81E-05	hsa-miR-6804-3p	3.54	2.8E-05
hsa-miR-6824-5p	3.63	2.54E-05	hsa-miR-6839-5p	5.22	1.33E-05
**Exclusively down-regulated in 25 μM CLZ Vs. control**					
hsa-miR-6851-3p	−5.96	0.0000	hsa-miR-6515-3p	−1.21	0.0015
hsa-miR-6785-3p	−6.52	0.0000	hsa-miR-4313	−1.24	0.0016
hsa-miR-6824-3p	−6.66	0.0001	hsa-miR-6069	−1.03	0.0020
hsa-miR-6756-3p	−5.45	0.0001	hsa-miR-6858-3p	−1.04	0.0030
hsa-miR-6819-3p	−1.18	0.0002	hsa-miR-1275	−1.36	0.0037
hsa-miR-4749-3p	−1.73	0.0002	hsa-miR-6870-3p	−1.23	0.0057
hsa-miR-4769-3p	−5.56	0.0002	hsa-miR-6880-3p	−6.03	0.0067
hsa-miR-1470	−5.43	0.0005	hsa-miR-1237-3p	−6.07	0.0088
hsa-miR-6812-3p	−5.56	0.0005	hsa-miR-33b-3p	−1.38	0.0120
hsa-miR-1281	−1.08	0.0006	hsa-let-7f-1-3p	−2.41	0.0426
hsa-miR-6737-3p	−1.17	0.0008	hsa-miR-6813-3p	−1.49	0.0456
hsa-miR-6132	−1.47	0.0009			
**Exclusively upregulated in 25 μM HLP + 25 μM CLZ Vs. control**			**Exclusively down regulated in 25μM HLP + 25μM CLZ Vs. control**		
hsa-miR-6759-3p	4.11	0.0004	hsa-miR-10a-5p	−4.27	0.0004
hsa-miR-6743-3p	4.56	0.0094	hsa-miR-29b-1-5p	−1.69	0.0110
hsa-miR-6834-3p	4.71	0.0132	hsa-miR-34a-3p	−4.31	0.0000
			hsa-miR-4532	−4.53	0.0000
			hsa-miR-7-1-3p	−4.50	0.0015

### Pathway Analysis of Differentially Expressed miRNAs

**FIGURE 4:**
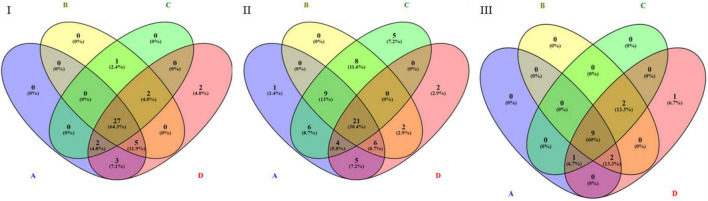
Venn diagram representing the distribution of upregulated miRNAs in various pharmacogenomic pathways. A – ABC transporters, B – drug metabolism cytochrome P450, C – drug metabolism other enzymes, D – metabolic pathways. **(I)** Upregulated in 25 μM HLP vs. control, **(II)** Upregulated in 25 μM CLZ vs. control, **(III)** Upregulated in 25 μM HLP + 25 μM CLZ vs. control.

**FIGURE 5:**
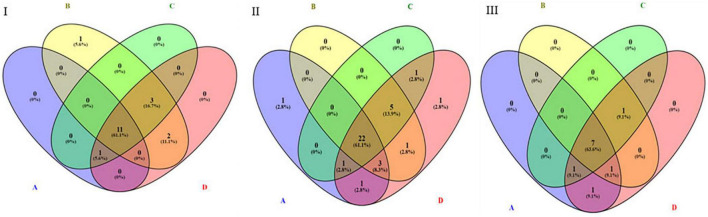
Venn diagram representing the distribution of downregulated miRNAs in various pharmacogenomic pathways. A – ABC transporters, B – drug metabolism cytochrome P450, C – drug metabolism other enzymes, D – metabolic pathways. **(I)** Downregulated in 25 μM HLP vs. control, **(II)** Downregulated in 25 μM CLZ vs. control, **(III)** Downregulated in 25 μM HLP + 25 μM CLZ vs. control.

By performing pathway analysis of differentially expressed miRNAs, we aimed to understand better the biological mechanisms regulated by the antipsychotic drug-induced miRNAs. The top biological pathways and the genes associated with miRNAs in each drug treatment condition are given in Table 4. Interestingly, among the upregulated miRNAs, there were several common genes that were targets of these miRNAs, but when considering the pathways, melanogenesis and opioid melanocortin seem to be the most common pathway influenced by these antipsychotic drugs. In contrast, among the downregulated miRNAs, there were rarely any overlapping target genes, and there was no any common pathway for these antipsychotic drug treatment groups. Further functional classification of differentially expressed miRNAs was performed with a focus on epigenetic and pharmacokinetic pathways including ABC transporters, drug metabolism cytochrome P450, drug metabolism other enzymes, and metabolic pathways. The number of miRNAs that target various genes in specific epigenetic and pharmacokinetic pathways is given in Table 5. The complete list of miRNAs belonging to epigenetic and pharmacokinetic functions is given in Supplementary Tables 2, 3, respectively. The four-way Venn diagram showing the distribution of upregulated miRNAs and downregulated miRNAs of each biological functional group in different treatment conditions is further represented in Figures 4, 5, respectively, depicting overlapping and exclusive to functional grouping.

**TABLE 4:** Pathway analysis of significantly differentially expressed miRNAs.

Pathway Category	Pathway	Genes
**Upregulated miRNAs in 25 μM HLP Vs. control**		
KEGG	TGF-beta signaling pathway	BMP4, ACVR2A, MAPK1, TNF, TGFBR1, LEFTY2, IFNG, DCN, SKP1, ACVR1C
KEGG	Melanogenesis	WNT10A, ADCY4, MAPK1, GNAO1, CREB3, MAP2K2, KIT, POMC, FZD4, CALM1
REACTOME	APC/C:Cdh1-mediated degradation of Skp2	PSMA1, PSMA5, PSMC2, UBC, UBE2D1, CDC26, PSMD9
REACTOME	Signaling by Wnt	PSMA1, PSMA5, PSMC2, PPP2R5C, UBC, SKP1, PSMD9
REACTOME	Phospho-APC/C mediated degradation of Cyclin A	PSMA1, PSMA5, PSMC2, UBC, UBE2D1, CDC26, PSMD9
PANTHER	Opioid proopiomelanocortin	GNGT2, GNAO1, VAMP1, POMC, SNAP25
**Upregulated miRNAs in 25 μM CLZ Vs. control**		
KEGG	Melanogenesis	WNT10A, ADCY4, MAPK1, PLCB3, WNT4, GNAO1, CREB3, MAP2K2, WNT3A, POMC
KEGG	Hedgehog signaling pathway	WNT10A, WNT4, CSNK1G2, WNT3A, GAS1, ZIC2, IHH
PANTHER	Synaptic vesicle trafficking	SYN2, SYT12, SYT6, SYT15, VAMP1, RIMS4
KEGG	Adipocytokine signaling pathway	TNF, ACSL1, LEPR, POMC, AKT3, CAMKK1, CAMKK2
PANTHER	Corticotropin releasing factor receptor signaling pathway	CRHR1, GNAL, GNGT2, VAMP1, POMC
KEGG	Prion diseases	MAPK1, MAP2K2, C5, ELK1, LAMC1
PANTHER	Opioid proopiomelanocortin	GNGT2, GNAO1, VAMP1, POMC, KCNK3
KEGG	Bladder cancer	E2F2, MAPK1, MAP2K2, RASSF1, MMP1
**Upregulated miRNAs in 25 μM HLP + 25 μM CLZ Vs. control**		
KEGG	Dilated cardiomyopathy	ADCY3, ADCY7, CACNG7, CACNG6, ITGA11, CACNB1, ITGA10, CACNA2D2
KEGG	Cardiac muscle contraction	ATP1B2, CACNG7, CACNG6, COX4I2, ATP1A3, CACNB1, CACNA2D2
KEGG	Arrhythmogenic right ventricular cardiomyopathy	CACNG7, CACNG6, ITGA11, CACNB1, ITGA10, CACNA2D2
KEGG	Antigen processing and presentation	IFNA21, PSME1, CD8B, TAP1, HLA-A, HLA-DQA2
REACTOME	Opioid Signaling	ADCY3, GNAL, GNGT2,ADCY7, PDE4C, PDYN
KEGG	Vibrio cholerae infection	ADCY3, ATP6AP1, ATP6V1E1, ATP6V0D1, ATP6V0B
KEGG	Hypertrophic cardiomyopathy	CACNG7, CACNG6, ITGA11, CACNB1, ITGA10, CACNA2D2
KEGG	MAPK signaling pathway	MAP2K2, CACNG7, CACNG6, CACNB1, TP53, ELK1,FGF13, MAPKAPK2, CACNA2D2, DUSP7
**Down regulated miRNAs in 25 μM HLP Vs. control**		
KEGG	Calcium signaling pathway	PRKCA, ADCY3, ADCY7, ADCY8, PPP3R1, PTGFR, VDAC1, HRH1, PLCG1, HTR7, PDE1A, PLCD3, ADRA1A, GNAS, PPP3CA, PRKACB, HTR2C, CACNA1D, ADRA1D
KEGG	GnRH signaling pathway	PRKCA, ADCY3, PLD1, ADCY7, ADCY8, MAPK10, NRAS, MAP3K3, GNAS, PRKACB, FSHB, CACNA1D, PLA2G2F
REACTOME	Processing of Capped Intron-Containing Pre-mRNA	PABPN1, POLR2E, SNRPD3, SNRPB2, POLR2I, SNRPD1, RNPS1, DDX23, PCBP1, GTF2F2, PCBP2, SRRM1, HNRNPH1, SNRPF
KEGG	Vibrio cholerae infection	PRKCA, ADCY3, ATP6V1A, PLCG1, GNAS, ATP6V1G1, PRKACB, KDELR1, ATP6V1F
KEGG	Vascular smooth muscle contraction	PRKCA, GNA13, ADCY3, PPP1CA, ADCY7, ADCY8, ADRA1A, GNAS, PRKACB, PPP1CC, CACNA1D, ADRA1D, PLA2G2F
KEGG	Long-term potentiation	PRKCA, NRAS, PPP1CA, ADCY8, CREBBP, PPP3R1, PPP3CA, PRKACB, PPP1CC
REACTOME	Influenza Infection	PABPN1, POLR2E, SNRPD3, POLR2I, SNRPD1, RNPS1, RPS6, RPS16, PCBP1, PCBP2, GTF2F2, RPL26L1, SRRM1, RPL37A, HNRNPH1, SNRPF
REACTOME	Opioid Signaling	ADCY3, PPP1CA, ADCY7, ADCY8, PDE4A, PDE1A, PPP3R1, GNB4, PPP3CA, PRKACB
KEGG	Pantothenate and CoA biosynthesis	BCAT1, COASY, ENPP3, DPYD
KEGG	Gap junction	PRKCA, ADCY3, NRAS, ADCY7, TUBB2B, ADCY8, TUBAL3, GNAS, PRKACB, HTR2C
PANTHER	Transcription regulation by bZIP transcription factor	TAF2, BRF1, POLR2E, C3ORF67, TAF8, GTF2F2, CREBBP, POLR2I
**Down regulated miRNAs in 25 μM CLZ Vs. control**		
KEGG	MAPK signaling pathway	TRAF2, IL1R1, FGFR3, ELK1, MAP3K6, BDNF, RASGRP3, IL1B, PRKACA, CACNG8, MAP2K3, RELB, FLNB, CACNA2D2, ARRB2, DUSP1, ARRB1, MAPK13, GADD45G, CACNA1G, MAPK8IP1, GADD45B, CRK, GADD45A, CACNA1D, MAP3K12
KEGG	Notch signaling pathway	CTBP1, NOTCH1, DLL4, DTX3, CREBBP, PTCRA, LFNG, NCOR2
**Down regulated miRNAs in 25 μM HLP + 25 μM CLZ Vs. control**		
PANTHER	Huntington disease	ARPC1A, TUBB2B, GRIK1, ACTR1A, GRIN1, CASP8, ACTR3C, WDR34, VAT1, NCOR2, KALRN
REACTOME	Cell Cycle Checkpoints	CDC7, CDC6, PSMC6, PSME2, HUS1, MDM2, ANAPC11, UBA52, PSMD8
REACTOME	Metabolism of lipids and lipoproteins	PNLIP, PPP1CA, BMP1, MVD, HSD3B7, SQLE, PRKACA, ACACB, PMVK, AGPAT3
REACTOME	Apoptosis	H1F0, PSMC6, XIAP, PSME2, BBC3, CASP7, CASP8, UBA52, PSMD8

**TABLE 5:** Functional classification of significantly differentially expressed miRNAs targeting epigenetic and pharmacokinetic pathways.

Pathways	25 μM HLP Upregulated	25 μM CLZ Upregulated	25 μM HLP + 25 μM CLZ Upregulated	25 μM HLP Downregulated	25 μM CLZ Downregulated	25 μM HLP + 25 μM CLZ Downregulated
**Epigenetic machinery**	13	20	6	9	13	4
**ABC transporters**	37	52	12	12	28	10
**Drug metabolism cytochrome P450**	35	46	13	17	31	9
**Drug metabolism other enzymes**	32	53	12	15	29	9
**Metabolic pathways**	41	40	15	17	35	11

## Discussion

Antipsychotic drugs are known to have a variable impact on patients and can induce several side effects which are known to be metabolic in nature. Keeping this in mind, this study was performed in the liver cell line as the first pass effect of any drug that undergoes in the liver. Therefore, it is important to understand how these antipsychotic drugs either in isolation or in combination impact the miRNA expression which in turn may impact metabolism. In a conventional clinical setting, it is difficult to evaluate the effect of antipsychotic drugs on miRNA expression for the following two major reasons: first, it is difficult to have completely drug naïve schizophrenia patients, and second, it is difficult to study the site of metabolism, i.e., liver of the patient to monitor the miRNA expression. Therefore, to resolve this issue to some extent, we used HepG2 cell lines to test how antipsychotic drugs would influence the miRNA expression in hepatic cells, which in turn might influence the drug metabolism. HepG2 cells have been widely used for drug toxicity and drug screening studies (Gerets et al., 2012). The most commonly used typical antipsychotic drug, haloperidol and atypical antipsychotic drugs, and clozapine were used at maximum tolerable dose concentration. From the clinical point of view, these concentrations used are therapeutically relevant, as these drugs are lipophilic; therefore, concentrations reaching hepatic tissue are 10–30 times higher than in serum (Raeder et al., 2006). In the majority of the studies, the *in vitro* efficacy of neuropsychiatric drugs is based only on acute drug administration (Bespalov et al., 2016). As per the prescription trends of antipsychotics, it is evident that prescribing high-dose antipsychotics and polypharmacy is common even in schizophrenia (Roh et al., 2015). Therefore, we suggest that the study design might reflect the human metabolic condition of patients with chronic schizophrenia. Haloperidol and clozapine were used in 25 μM concentration as this concentration was found to be the effective dose at which gene expression and epigenetic modifications were reported in a previous *in vitro* study (Swathy et al., 2018).

Results of miRNA profiling studies provide evidence that several miRNAs are altered on antipsychotic drug treatment signifying the role of miRNAs in antipsychotic drug response. Several miRNAs were found to be exclusively altered in certain drug treatment conditions indicating that these miRNAs are specific signatures of the particular drug. The differentially expressed miRNAs were predicted to target genes relevant in various pathways including mainly inflammatory, neuronal, and metabolic pathways. The influence of the immune system in schizophrenia and the effects of antipsychotic drugs on the inflammatory response system of patients with schizophrenia has been reported (Al-Amin et al., 2013; Srinivas et al., 2016). Considering the pharmacodynamics and pharmacokinetic actions of the drug, it is obvious that miRNAs target genes belonging to the neuronal and metabolic pathways. This was further evident in our earlier observation where it has been demonstrated that antipsychotics do impact various neurotransmitter gene expression through miRNA modulation (Swathy and Banerjee, 2017a). Drugs can also directly act on the epigenetic genes to modify the activity of epigenetic proteins and thus influence the epigenetic signatures. Antipsychotic drugs were reported to influence DNA methyltransferase expression resulting in global hypermethylation through modulation of miRNA miR-29b (Swathy et al., 2018). In postmortem brains of patients with chronic schizophrenia, an increase in DNA-methyltransferase 1 (DNMT1), ten-eleven methylcytosine dioxygenase 1 (TET1), and enrichment of 5-methylcytosine (5MC) and 5-hydroxymethylcytosine (5HMC) at neocortical GABAergic and glutamatergic gene promoters were reported. Identification of several miRNAs that targets the epigenetic machinery in this study further strengthens our earlier observation that global methylation profile might be impacted by antipsychotic treatment through miRNA modulation. Antipsychotic drugs were reported to influence the expression of ABCB1, CYP1A2, and CYP3A4 through miRNA (Swathy et al., 2017). Identification of several miRNAs through global profiling, targeting key pharmacokinetic pathways such as ABC transporters and drug metabolism, further strengthens that antipsychotic drugs can impact pharmacokinetic parameters through miRNA alterations. Studies on metabolic side effects have been reported for psychotic drugs through miRNA modulation (Grajales et al., 2019; Carli et al., 2021). Moreover, antipsychotic drugs can also protect neuronal cells from neurotoxicity by controlling the miRNA-based pathway (Zhu et al., 2016; Yu et al., 2018). Even our earlier *in silico* prediction using Pharmaco-miR identified that antipsychotic drugs can target a large number of miRNAs, and this study further validates the observation. Alteration of genes in metabolic pathways indicates that miRNAs could play a role in metabolic side effects associated with antipsychotic drugs. It is also suggested that miRNA profile-based biomarkers can also be considered as an alternative to genetic biomarkers to analyze treatment responsiveness.

Several miRNAs have been reported to be dysregulated in schizophrenia (Caputo et al., 2015). In schizophrenia, miR-181b, let-7d, and miR-219 have been reported to be upregulated, whereas miR-664a-3p and miR-339 are reported to be downregulated (Beveridge and Cairns, 2012). Our study shows that haloperidol treatment induced upregulation of miR-181b and miR-219 (< 5-fold). Therefore, if one has to believe that upregulated signatures are real indicators of pathogenesis, then on antipsychotic treatment, the expression is regulated through miRNA. But, in sharp contrast, the miRNAs that were reported to be downregulated in schizophrenia such as miR-664a-3p and miR-339 were also downregulated by haloperidol, clozapine, and even in combination treatment. These points to the possibility that several miRNAs reported to be dysregulated in schizophrenia could be mired by antipsychotic drugs. This needs a careful review.

In conclusion, our study provides evidence that antipsychotic drugs induce the differential expression of various miRNAs that target genes relevant in various pathways including epigenetic and pharmacokinetic pathways in the liver cell line which is the target for drug metabolism. This study also highlights the possibility that earlier reports on miRNA alteration in schizophrenia might not be the direct indicator of pathogenesis but might be an indicator of drug-mediated response as observed in this study.

## Data Availability Statement

The datasets presented in this study can be found in online repositories. The names of the repository/repositories and accession number(s) can be found below: ArrayExpress, accession E-MTAB-11458.

## Author Contributions

SB and MB conceptualized this study, interpreted and wrote the manuscript. SB performed the work and analysis. Both authors contributed to the article and approved the submitted version.

## Conflict of Interest

The authors declare that the research was conducted in the absence of any commercial or financial relationships that could be construed as a potential conflict of interest.

## Publisher’s Note

All claims expressed in this article are solely those of the authors and do not necessarily represent those of their affiliated organizations, or those of the publisher, the editors and the reviewers. Any product that may be evaluated in this article, or claim that may be made by its manufacturer, is not guaranteed or endorsed by the publisher.

## Footnotes

1
bioinfogp.cnb.csic.es/tools/

2
david.ncifcrf.gov/home.jsp

3
mirwalk.umm.uni-heidelberg.de/
