# Supplementary material for: Understanding Pharmaco-Epigenomic Response of Antipsychotic Drugs Using Genome-Wide MicroRNA Expression Profile in Liver Cell Line
Source: Front Mol Neurosci. 2022 Mar 22;15:786632. doi: 10.3389/fnmol.2022.786632 (PMC8980709; doi:10.3389/fnmol.2022.786632)
Supplement: Supplementary file 1 [file Table_1.DOCX]

**Supplementary Table 1. List of differentially expressed miRNAs above threshold foldchange upon antipsychotic treatment**

| **List of differentially expressed miRNAs in 25 µM HLP Vs Control** | | | | | |
| --- | --- | --- | --- | --- | --- |
| **Upregulated** | **FC** | **P value** | **Downregulated** | **FC** | **P value** |
| hsa-miR-224-3p | 4.07 | 0 | hsa-miR-1908-3p | -4.73 | 0 |
| hsa-miR-4254 | 5.24 | 0 | hsa-miR-6752-3p | -4.87 | 0 |
| hsa-miR-619-5p | 3.02 | 0 | hsa-miR-664a-3p | -4.67 | 0.0001 |
| hsa-miR-1288-3p | 5.62 | 0 | hsa-miR-6798-3p | -4.88 | 0.0001 |
| hsa-miR-4419a | 5.22 | 0 | hsa-miR-7114-3p | -4.70 | 0.0001 |
| hsa-miR-6807-5p | 5.83 | 0.0001 | hsa-miR-6757-3p | -4.71 | 0.0001 |
| hsa-miR-130a-3p | 5.16 | 0.0001 | hsa-miR-6789-5p | -4.96 | 0.0002 |
| hsa-miR-6512-5p | 5.26 | 0.0001 | hsa-miR-371b-5p | -6.11 | 0.0003 |
| hsa-miR-6833-5p | 4.90 | 0.0001 | hsa-miR-7974 | -4.67 | 0.0003 |
| hsa-miR-340-5p | 5.53 | 0.0001 | hsa-miR-5571-5p | -4.73 | 0.0007 |
| hsa-miR-378d | 4.54 | 0.0001 | hsa-miR-4787-5p | -1.31 | 0.0029 |
| hsa-miR-374c-5p | 3.98 | 0.0002 | hsa-miR-6068 | -1.19 | 0.0035 |
| hsa-miR-5699-5p | 3.77 | 0.0002 | hsa-miR-6763-3p | -1.69 | 0.0041 |
| hsa-miR-7152-3p | 5.55 | 0.0003 | hsa-miR-6777-3p | -5.27 | 0.0067 |
| hsa-miR-3194-5p | 3.75 | 0.0003 | hsa-miR-6848-3p | -5.29 | 0.0079 |
| hsa-miR-4521 | 4.26 | 0.0003 | hsa-let-7b-3p | -2.34 | 0.0083 |
| hsa-miR-454-3p | 4.36 | 0.0003 | hsa-miR-4758-3p | -5.15 | 0.0084 |
| hsa-miR-421 | 4.18 | 0.0003 | hsa-miR-4436b-5p | -5.29 | 0.0084 |
| hsa-miR-181b-5p | 3.70 | 0.0004 | hsa-miR-4652-3p | -4.68 | 0.0118 |
| hsa-miR-5088-5p | 5.83 | 0.0004 | hsa-miR-3613-3p | -5.25 | 0.0126 |
| hsa-miR-892b | 4.92 | 0.0005 | hsa-let-7f-1-3p | -4.25 | 0.1269 |
| hsa-miR-505-5p | 3.99 | 0.0005 | hsa-miR-425-3p | -3.93 | 0.1983 |
| hsa-miR-361-3p | 4.19 | 0.0006 | hsa-miR-1539 | -3.75 | 0.2123 |
| hsa-miR-98-5p | 4.73 | 0.0006 | hsa-miR-1237-3p | -1.02 | 0.2166 |
| hsa-miR-6794-5p | 5.23 | 0.0006 | hsa-miR-6851-3p | -3.33 | 0.2248 |
| hsa-miR-3156-5p | 5.85 | 0.0007 | hsa-miR-6785-3p | -3.66 | 0.2302 |
| hsa-miR-22-5p | 3.82 | 0.001 | hsa-miR-6824-3p | -3.76 | 0.233 |
| hsa-miR-200b-5p | 3.04 | 0.0011 | hsa-miR-1229-5p | -3.57 | 0.2378 |
| hsa-miR-6780a-5p | 5.10 | 0.0011 | hsa-miR-6880-3p | -3.21 | 0.2815 |
| hsa-miR-335-5p | 3.69 | 0.0013 | hsa-miR-6760-3p | -3.21 | 0.2832 |
| hsa-miR-340-3p | 3.82 | 0.0013 |  |  |  |
| hsa-miR-221-5p | 4.03 | 0.0017 |  |  |  |
| hsa-miR-181d-5p | 4.10 | 0.0018 |  |  |  |
| hsa-miR-4298 | 3.77 | 0.0019 |  |  |  |
| hsa-miR-3127-5p | 5.37 | 0.005 |  |  |  |
| hsa-miR-4800-5p | 4.30 | 0.0085 |  |  |  |
| hsa-miR-219a-5p | 4.55 | 0.0092 |  |  |  |
| hsa-miR-452-5p | 4.39 | 0.0099 |  |  |  |
| hsa-miR-4701-3p | 4.64 | 0.011 |  |  |  |
| hsa-miR-200a-5p | 3.77 | 0.0128 |  |  |  |
| hsa-miR-378a-5p | 4.34 | 0.0135 |  |  |  |
| hsa-miR-3141 | 4.43 | 0.0182 |  |  |  |

| **List of upregulated miRNAs in 25 µM CLZ Vs Control** | | | | | |
| --- | --- | --- | --- | --- | --- |
| **Upregulated** | **FC** | **P value** | **Upregulated** | **FC** | **P value** |
| hsa-miR-7152-3p | 5.56 | 0 | hsa-miR-4298 | 3.53 | 0.0004 |
| hsa-miR-1288-3p | 5.24 | 0 | hsa-miR-221-5p | 3.58 | 0.0005 |
| hsa-miR-6839-5p | 5.23 | 0 | hsa-miR-4462 | 4.07 | 0.0005 |
| hsa-miR-6076 | 5.18 | 0 | hsa-miR-2392 | 4.15 | 0.0005 |
| hsa-miR-6824-5p | 3.63 | 0 | hsa-miR-340-5p | 5.13 | 0.0005 |
| hsa-miR-150-3p | 5.55 | 0 | hsa-miR-340-5p | 5.13 | 0.0005 |
| hsa-miR-6804-3p | 3.54 | 0 | hsa-miR-4633-5p | 3.63 | 0.0006 |
| hsa-miR-4257 | 4.17 | 0 | hsa-miR-181b-5p | 4.13 | 0.0006 |
| hsa-miR-4499 | 4.80 | 0 | hsa-miR-22-5p | 3.65 | 0.0006 |
| hsa-miR-3156-5p | 5.30 | 0 | hsa-miR-98-5p | 4.33 | 0.0007 |
| hsa-miR-454-3p | 4.26 | 0 | hsa-miR-4701-3p | 5.95 | 0.0007 |
| hsa-miR-181d-5p | 4.95 | 0 | hsa-miR-6780a-5p | 4.77 | 0.0007 |
| hsa-miR-219a-5p | 5.25 | 0 | hsa-miR-33a-5p | 4.59 | 0.0008 |
| hsa-miR-1236-5p | 4.57 | 0.0001 | hsa-miR-4521 | 3.65 | 0.0008 |
| hsa-miR-6794-5p | 5.17 | 0.0001 | hsa-miR-5088-5p | 5.68 | 0.0008 |
| hsa-miR-629-3p | 4.94 | 0.0001 | hsa-miR-421 | 3.75 | 0.0009 |
| hsa-miR-4419a | 4.74 | 0.0001 | hsa-miR-6801-3p | 3.59 | 0.0011 |
| hsa-miR-6512-5p | 4.87 | 0.0001 | hsa-miR-224-3p | 3.69 | 0.0013 |
| hsa-miR-6768-5p | 4.22 | 0.0001 | hsa-miR-6775-5p | 3.77 | 0.0014 |
| hsa-miR-892b | 4.78 | 0.0001 | hsa-miR-6741-5p | 3.42 | 0.0017 |
| hsa-miR-6807-5p | 5.79 | 0.0001 | hsa-miR-4254 | 5.01 | 0.0022 |
| hsa-miR-5699-5p | 4.70 | 0.0001 | hsa-miR-378b | 3.64 | 0.0024 |
| hsa-miR-3127-5p | 5.17 | 0.0001 | hsa-miR-200b-5p | 2.54 | 0.0026 |
| hsa-miR-4327 | 4.83 | 0.0001 | hsa-miR-4651 | 2.40 | 0.0046 |
| hsa-miR-134-5p | 4.99 | 0.0001 | hsa-miR-130a-3p | 4.16 | 0.0072 |
| hsa-miR-550a-3-5p | 3.49 | 0.0001 | hsa-miR-183-3p | 2.69 | 0.0075 |
| hsa-miR-4481 | 3.42 | 0.0002 | hsa-miR-3648 | 4.23 | 0.0084 |
| hsa-miR-328-5p | 3.66 | 0.0002 | hsa-miR-505-5p | 3.19 | 0.0086 |
| hsa-miR-1224-5p | 4.77 | 0.0002 | hsa-let-7d-3p | 2.52 | 0.0094 |
| hsa-miR-361-3p | 4.11 | 0.0002 | hsa-miR-6786-5p | 3.95 | 0.0099 |
| hsa-miR-6833-5p | 4.85 | 0.0002 | hsa-miR-378a-5p | 4.11 | 0.0117 |
| hsa-miR-340-3p | 3.47 | 0.0002 | hsa-miR-3194-5p | 4.07 | 0.0124 |
| hsa-miR-1469 | 3.52 | 0.0003 | hsa-miR-5195-3p | 3.99 | 0.0142 |
| hsa-miR-4270 | 5.06 | 0.0003 | hsa-miR-202-3p | 4.01 | 0.0223 |
| hsa-miR-378d | 4.39 | 0.0003 | hsa-miR-135a-3p | 2.41 | 0.035 |
| hsa-miR-3141 | 5.05 | 0.0003 |  |  |  |
| **List of down regulated miRNAs in 25 µM CLZ Vs Control** | | | | | |
| **Downregulated** | **FC** | **P value** | **Downregulated** | **FC** | **P value** |
| hsa-miR-6851-3p | -5.96 | 0.0000 | hsa-miR-6737-3p | -1.17 | 0.0008 |
| hsa-miR-1908-3p | -5.43 | 0.0000 | hsa-miR-6132 | -1.47 | 0.0009 |
| hsa-miR-339-5p | -4.62 | 0.0000 | hsa-miR-6515-3p | -1.21 | 0.0015 |
| hsa-miR-6785-3p | -6.52 | 0.0000 | hsa-miR-4313 | -1.24 | 0.0016 |
| hsa-miR-6824-3p | -6.66 | 0.0001 | hsa-miR-6069 | -1.03 | 0.0020 |
| hsa-miR-6798-3p | -5.57 | 0.0001 | hsa-miR-6858-3p | -1.04 | 0.0030 |
| hsa-miR-7114-3p | -5.39 | 0.0001 | hsa-miR-1275 | -1.36 | 0.0037 |
| hsa-miR-6757-3p | -5.40 | 0.0001 | hsa-miR-6777-3p | -5.96 | 0.0052 |
| hsa-miR-6756-3p | -5.45 | 0.0001 | hsa-miR-6870-3p | -1.23 | 0.0057 |
| hsa-miR-6819-3p | -1.18 | 0.0002 | hsa-miR-6848-3p | -5.98 | 0.0062 |
| hsa-miR-4749-3p | -1.73 | 0.0002 | hsa-miR-4758-3p | -5.84 | 0.0065 |
| hsa-miR-4769-3p | -5.56 | 0.0002 | hsa-miR-6880-3p | -6.03 | 0.0067 |
| hsa-miR-6763-3p | -6.71 | 0.0003 | hsa-miR-1237-3p | -6.07 | 0.0088 |
| hsa-miR-7974 | -5.37 | 0.0003 | hsa-miR-4652-3p | -5.37 | 0.0090 |
| hsa-miR-1470 | -5.43 | 0.0005 | hsa-miR-3613-3p | -5.95 | 0.0099 |
| hsa-miR-6812-3p | -5.56 | 0.0005 | hsa-miR-33b-3p | -1.38 | 0.0120 |
| hsa-miR-5571-5p | -5.43 | 0.0006 | hsa-let-7f-1-3p | -2.41 | 0.0426 |
| hsa-miR-1281 | -1.08 | 0.0006 | hsa-miR-6813-3p | -1.49 | 0.0456 |
| **List of differentially expressed miRNAs in 25 µM HLP + 25 µM CLZ Vs control** | | | | | |
| **Upregulated** | **FC** | **P value** | **Downregulated** | **FC** | **P value** |
| hsa-miR-7152-3p | 5.44 | 0 | hsa-miR-339-5p | -3.45 | 0.0000 |
| hsa-miR-6794-5p | 5.66 | 0 | hsa-miR-34a-3p | -4.31 | 0.0000 |
| hsa-miR-6807-5p | 5.68 | 0 | hsa-miR-4532 | -4.53 | 0.0000 |
| hsa-miR-5088-5p | 5.41 | 0.0001 | hsa-miR-664a-3p | -4.20 | 0.0001 |
| hsa-miR-150-3p | 4.16 | 0.0001 | hsa-miR-7974 | -4.20 | 0.0004 |
| hsa-miR-1288-3p | 5.30 | 0.0002 | hsa-miR-10a-5p | -4.27 | 0.0004 |
| hsa-miR-629-3p | 3.63 | 0.0002 | hsa-miR-7-1-3p | -4.50 | 0.0015 |
| hsa-miR-4419a | 4.10 | 0.0002 | hsa-miR-6848-3p | -4.81 | 0.0095 |
| hsa-miR-3141 | 4.17 | 0.0003 | hsa-miR-4758-3p | -4.67 | 0.0101 |
| hsa-miR-3156-5p | 5.59 | 0.0004 | hsa-miR-29b-1-5p | -1.69 | 0.0110 |
| hsa-miR-6759-3p | 4.11 | 0.0004 | hsa-miR-371b-5p | -1.18 | 0.0119 |
| hsa-miR-4633-5p | 4.21 | 0.0016 |  |  |  |
| hsa-miR-6743-3p | 4.56 | 0.0094 |  |  |  |
| hsa-miR-4270 | 4.61 | 0.0112 |  |  |  |
| hsa-miR-6834-3p | 4.71 | 0.0132 |  |  |  |

**Supplementary Table 2. List of differentially expressed miRNAs targeting epigenetic genes upon antipsychotic drug treatment**

| **Gene** | **25µM HLP Vs Control** | | **25µM CLZ Vs Control** | | **25µM HLP +25µM CLZ**  **Vs Control** | |
| --- | --- | --- | --- | --- | --- | --- |
|  | **Up**  **regulated** | **Down**  **regulated** | **Up**  **regulated** | **Down**  **regulated** | **Up**  **regulated** | **Down**  **regulated** |
| *DNMT1* | miR-130a-3p | miR-6777-3p | miR-130a-3p | miR-6777-3p |  | miR-29b |
| *DNMT3A* | miR-505-5p, miR-221-5p,  miR-361-3p,  miR-1288-3p | miR-6752-3p | miR-505-5p, miR-221-5p, miR-4633-5p, miR-361-3p, miR-4270, miR-6839-5p | miR-4313, miR-1470, miR-4769-3p, miR-3613-3p | miR-4633-5p,  miR-4270,  miR-1288-3p | miR-29b |
| *DNMT3B* |  | miR-7114-3p | miR-6801-3p, miR-629-3p | miR-1275, miR-6132, miR-339-5p, miR-7114-3p | miR-629-3p | miR-339-5p,  miR-29b |
| *MBD2* | miR-221-5p | let-7b-3p,  miR-5571-5p,  miR-6752-3p | miR-221-5p | miR-5571-5p |  |  |
| *MECP2* | miR-421 | miR-4436b-5p | miR-4481, miR-421,  miR-2392, miR-4257, miR-4254, miR-5088-5p | miR-1281, miR-6737-3p, miR-1275, miR-6132, miR-339-5p, miR-1237-3p | miR-5088-5p | miR-339-5p,  miR-7-1-3p |
| *TET1* | miR-452-5p, miR-4701-3p |  | miR-4701-3p |  |  |  |
| *TET2* | miR-181b-5p,  miR-505-5p,  miR-181d-5p,  miR-452-5p | let-7b-3p, miR-664a-3p | miR-505-5p, miR-181b-5p, miR-181d-5p |  | miR-6759-3p | miR-664a-3p |
| *TET3* |  |  | miR-6786-5p | miR-4749-3p,  miR-6756-3p |  |  |

**Supplementary Table 3: List of differentially expressed miRNAs targeting pharmacokinetic pathways genes upon antipsychotic drug treatment**

| **25µM HLP Vs Control (upregulated)** | | | | | |
| --- | --- | --- | --- | --- | --- |
| **ABC transporters** | **Drug metabolism cytochrome P450** | **Drug metabolism other enzymes** | | **Metabolic pathways** | |
| hsa-miR-619-5p | hsa-miR-619-5p | hsa-miR-619-5p | | hsa-miR-224-3p | |
| hsa-miR-200b-5p | hsa-miR-181b-5p | hsa-miR-200b-5p | | hsa-miR-4254 | |
| hsa-miR-181b-5p | hsa-miR-3194-5p | hsa-miR-181b-5p | | hsa-miR-619-5p | |
| hsa-miR-3194-5p | hsa-miR-5699-5p | hsa-miR-3194-5p | | hsa-miR-1288-3p | |
| hsa-miR-5699-5p | hsa-miR-4298 | hsa-miR-5699-5p | | hsa-miR-4419a | |
| hsa-miR-4298 | hsa-miR-22-5p | hsa-miR-200a-5p | | hsa-miR-6807-5p | |
| hsa-miR-200a-5p | hsa-miR-374c-5p | hsa-miR-22-5p | | hsa-miR-130a-3p | |
| hsa-miR-340-3p | hsa-miR-505-5p | hsa-miR-374c-5p | | hsa-miR-6512-5p | |
| hsa-miR-22-5p | hsa-miR-221-5p | hsa-miR-505-5p | | hsa-miR-6833-5p | |
| hsa-miR-374c-5p | hsa-miR-224-3p | hsa-miR-224-3p | | hsa-miR-340-5p | |
| hsa-miR-505-5p | hsa-miR-181d-5p | hsa-miR-181d-5p | | hsa-miR-378d | |
| hsa-miR-221-5p | hsa-miR-421 | hsa-miR-421 | | hsa-miR-374c-5p | |
| hsa-miR-224-3p | hsa-miR-361-3p | hsa-miR-361-3p | | hsa-miR-5699-5p | |
| hsa-miR-181d-5p | hsa-miR-4800-5p | hsa-miR-4800-5p | | hsa-miR-7152-3p | |
| hsa-miR-421 | hsa-miR-378a-5p | hsa-miR-378a-5p | | hsa-miR-3194-5p | |
| hsa-miR-361-3p | hsa-miR-454-3p | hsa-miR-454-3p | | hsa-miR-4521 | |
| hsa-miR-4521 | hsa-miR-452-5p | hsa-miR-452-5p | | hsa-miR-454-3p | |
| hsa-miR-378a-5p | hsa-miR-3141 | hsa-miR-3141 | | hsa-miR-421 | |
| hsa-miR-454-3p | hsa-miR-378d | hsa-miR-219a-5p | | hsa-miR-181b-5p | |
| hsa-miR-3141 | hsa-miR-219a-5p | hsa-miR-98-5p | | hsa-miR-5088-5p | |
| hsa-miR-378d | hsa-miR-98-5p | hsa-miR-6780a-5p | | hsa-miR-892b | |
| hsa-miR-219a-5p | hsa-miR-6833-5p | hsa-miR-130a-3p | | hsa-miR-505-5p | |
| hsa-miR-4701-3p | hsa-miR-6780a-5p | hsa-miR-6794-5p | | hsa-miR-361-3p | |
| hsa-miR-98-5p | hsa-miR-130a-3p | hsa-miR-4254 | | hsa-miR-98-5p | |
| hsa-miR-6833-5p | hsa-miR-4419a | hsa-miR-6512-5p | | hsa-miR-6794-5p | |
| hsa-miR-6780a-5p | hsa-miR-6794-5p | hsa-miR-3127-5p | | hsa-miR-3156-5p | |
| hsa-miR-130a-3p | hsa-miR-4254 | hsa-miR-340-5p | | hsa-miR-22-5p | |
| hsa-miR-4419a | hsa-miR-6512-5p | hsa-miR-7152-3p | | hsa-miR-200b-5p | |
| hsa-miR-4254 | hsa-miR-3127-5p | hsa-miR-1288-3p | | hsa-miR-6780a-5p | |
| hsa-miR-6512-5p | hsa-miR-340-5p | hsa-miR-6807-5p | | hsa-miR-335-5p | |
| hsa-miR-3127-5p | hsa-miR-7152-3p | hsa-miR-5088-5p | | hsa-miR-340-3p | |
| hsa-miR-340-5p | hsa-miR-1288-3p | hsa-miR-3156-5p | | hsa-miR-221-5p | |
| hsa-miR-7152-3p | hsa-miR-6807-5p |  | | hsa-miR-181d-5p | |
| hsa-miR-1288-3p | hsa-miR-5088-5p |  | | hsa-miR-4298 | |
| hsa-miR-6807-5p | hsa-miR-3156-5p |  | | hsa-miR-3127-5p | |
| hsa-miR-5088-5p |  |  | | hsa-miR-219a-5p | |
| hsa-miR-3156-5p |  |  | | hsa-miR-452-5p | |
|  |  |  | | hsa-miR-4701-3p | |
|  |  |  | | hsa-miR-200a-5p | |
|  |  |  | | hsa-miR-378a-5p | |
|  |  |  | | hsa-miR-3141 | |
| **25µM HLP Vs Control (downregulated)** | | | | | |
| **ABC transporters** | **Drug metabolism cytochrome P450** | | **Drug metabolism other enzymes** | | **Metabolic pathways** |
| hsa-miR-6752-3p | hsa-miR-1908-3p | | hsa-miR-1908-3p | | hsa-miR-1908-3p |
| hsa-miR-664a-3p | hsa-miR-6752-3p | | hsa-miR-6752-3p | | hsa-miR-6752-3p |
| hsa-miR-7114-3p | hsa-miR-664a-3p | | hsa-miR-664a-3p | | hsa-miR-664a-3p |
| hsa-miR-6757-3p | hsa-miR-6798-3p | | hsa-miR-7114-3p | | hsa-miR-6798-3p |
| hsa-miR-371b-5p | hsa-miR-7114-3p | | hsa-miR-6757-3p | | hsa-miR-7114-3p |
| hsa-miR-7974 | hsa-miR-6757-3p | | hsa-miR-6789-5p | | hsa-miR-6757-3p |
| hsa-miR-5571-5p | hsa-miR-6789-5p | | hsa-miR-371b-5p | | hsa-miR-6789-5p |
| hsa-miR-6763-3p | hsa-miR-371b-5p | | hsa-miR-7974 | | hsa-miR-371b-5p |
| hsa-miR-6777-3p | hsa-miR-7974 | | hsa-miR-5571-5p | | hsa-miR-7974 |
| hsa-miR-6848-3p | hsa-miR-5571-5p | | hsa-miR-6763-3p | | hsa-miR-5571-5p |
| hsa-let-7b-3p | hsa-miR-4787-5p | | hsa-miR-6777-3p | | hsa-miR-4787-5p |
| hsa-miR-4758-3p | hsa-miR-6068 | | hsa-miR-6848-3p | | hsa-miR-6763-3p |
|  | hsa-miR-6763-3p | | hsa-let-7b-3p | | hsa-miR-6777-3p |
|  | hsa-miR-6777-3p | | hsa-miR-4758-3p | | hsa-miR-6848-3p |
|  | hsa-let-7b-3p | | hsa-miR-4436b-5p | | hsa-let-7b-3p |
|  | hsa-miR-4758-3p | |  | | hsa-miR-4758-3p |
|  | hsa-miR-4436b-5p | |  | | hsa-miR-4436b-5p |
| **25µM CLZ Vs Control (upregulated)** | | | | | |
| **ABC transporters** | **Drug metabolism CYP P450** | | **Drug metabolism other enzymes** | | **Metabolic pathways** |
| hsa-miR-4651 | hsa-miR-183-3p | | hsa-miR-200b-5p | | hsa-miR-4651 |
| hsa-let-7d-3p | hsa-miR-505-5p | | hsa-miR-183-3p | | hsa-miR-135a-3p |
| hsa-miR-200b-5p | hsa-miR-4481 | | hsa-miR-505-5p | | hsa-let-7d-3p |
| hsa-miR-183-3p | hsa-miR-6741-5p | | hsa-miR-4481 | | hsa-miR-200b-5p |
| hsa-miR-505-5p | hsa-miR-550a-3-5p | | hsa-miR-550a-3-5p | | hsa-miR-183-3p |
| hsa-miR-4481 | hsa-miR-4298 | | hsa-miR-6801-3p | | hsa-miR-505-5p |
| hsa-miR-6741-5p | hsa-miR-6804-3p | | hsa-miR-4633-5p | | hsa-miR-4481 |
| hsa-miR-340-3p | hsa-miR-221-5p | | hsa-miR-6824-5p | | hsa-miR-6741-5p |
| hsa-miR-550a-3-5p | hsa-miR-4633-5p | | hsa-miR-22-5p | | hsa-miR-340-3p |
| hsa-miR-1469 | hsa-miR-6824-5p | | hsa-miR-328-5p | | hsa-miR-550a-3-5p |
| hsa-miR-4298 | hsa-miR-378b | | hsa-miR-224-3p | | hsa-miR-4298 |
| hsa-miR-221-5p | hsa-miR-22-5p | | hsa-miR-421 | | hsa-miR-6804-3p |
| hsa-miR-6801-3p | hsa-miR-224-3p | | hsa-miR-6786-5p | | hsa-miR-221-5p |
| hsa-miR-4633-5p | hsa-miR-421 | | hsa-miR-5195-3p | | hsa-miR-6801-3p |
| hsa-miR-6824-5p | hsa-miR-6775-5p | | hsa-miR-202-3p | | hsa-miR-4633-5p |
| hsa-miR-378b | hsa-miR-5195-3p | | hsa-miR-3194-5p | | hsa-miR-6824-5p |
| hsa-miR-22-5p | hsa-miR-202-3p | | hsa-miR-361-3p | | hsa-miR-378b |
| hsa-miR-4521 | hsa-miR-3194-5p | | hsa-miR-378a-5p | | hsa-miR-22-5p |
| hsa-miR-328-5p | hsa-miR-361-3p | | hsa-miR-181b-5p | | hsa-miR-4521 |
| hsa-miR-224-3p | hsa-miR-378a-5p | | hsa-miR-130a-3p | | hsa-miR-328-5p |
| hsa-miR-421 | hsa-miR-181b-5p | | hsa-miR-4257 | | hsa-miR-224-3p |
| hsa-miR-6786-5p | hsa-miR-130a-3p | | hsa-miR-6768-5p | | hsa-miR-421 |
| hsa-miR-5195-3p | hsa-miR-4257 | | hsa-miR-454-3p | | hsa-miR-6775-5p |
| hsa-miR-202-3p | hsa-miR-6768-5p | | hsa-miR-98-5p | | hsa-miR-6786-5p |
| hsa-miR-3194-5p | hsa-miR-454-3p | | hsa-miR-1236-5p | | hsa-miR-5195-3p |
| hsa-miR-361-3p | hsa-miR-98-5p | | hsa-miR-5699-5p | | hsa-miR-202-3p |
| hsa-miR-378a-5p | hsa-miR-378d | | hsa-miR-6780a-5p | | hsa-miR-3194-5p |
| hsa-miR-181b-5p | hsa-miR-1236-5p | | hsa-miR-4499 | | hsa-miR-361-3p |
| hsa-miR-2392 | hsa-miR-33a-5p | | hsa-miR-6512-5p | | hsa-miR-378a-5p |
| hsa-miR-130a-3p | hsa-miR-5699-5p | | hsa-miR-629-3p | | hsa-miR-181b-5p |
| hsa-miR-4257 | hsa-miR-4419a | | hsa-miR-181d-5p | | hsa-miR-2392 |
| hsa-miR-6768-5p | hsa-miR-6780a-5p | | hsa-miR-134-5p | | hsa-miR-130a-3p |
| hsa-miR-454-3p | hsa-miR-1224-5p | | hsa-miR-4254 | | hsa-miR-4257 |
| hsa-miR-98-5p | hsa-miR-4499 | | hsa-miR-3141 | | hsa-miR-6768-5p |
| hsa-miR-378d | hsa-miR-6833-5p | | hsa-miR-4270 | | hsa-miR-3648 |
| hsa-miR-1236-5p | hsa-miR-6512-5p | | hsa-miR-340-5p | | hsa-miR-454-3p |
| hsa-miR-33a-5p | hsa-miR-181d-5p | | hsa-miR-3127-5p | | hsa-miR-98-5p |
| hsa-miR-5699-5p | hsa-miR-134-5p | | hsa-miR-6794-5p | | hsa-miR-378d |
| hsa-miR-4419a | hsa-miR-4254 | | hsa-miR-6076 | | hsa-miR-1236-5p |
| hsa-miR-6780a-5p | hsa-miR-3141 | | hsa-miR-6839-5p | | hsa-miR-33a-5p |
| hsa-miR-4270 | hsa-miR-1288-3p | | hsa-miR-5699-5p | |  |
| hsa-miR-340-5p | hsa-miR-219a-5p | | hsa-miR-4419a | |  |
| hsa-miR-3127-5p | hsa-miR-3156-5p | | hsa-miR-6780a-5p | |  |
| hsa-miR-6794-5p | hsa-miR-7152-3p | | hsa-miR-1224-5p | |  |
| hsa-miR-6076 | hsa-miR-5088-5p | | hsa-miR-892b | |  |
| hsa-miR-1288-3p | hsa-miR-6807-5p | | hsa-miR-4499 | |  |
| hsa-miR-219a-5p |  | | hsa-miR-4327 | |  |
| hsa-miR-3156-5p |  | | hsa-miR-6833-5p | |  |
| hsa-miR-150-3p |  | | hsa-miR-6512-5p | |  |
| hsa-miR-7152-3p |  | | hsa-miR-629-3p | |  |
| hsa-miR-5088-5p |  | | hsa-miR-181d-5p | |  |
| hsa-miR-6807-5p |  | | hsa-miR-134-5p | |  |
|  |  | | hsa-miR-4254 | |  |
|  |  | | hsa-miR-3141 | |  |
|  |  | | hsa-miR-4270 | |  |
|  |  | | hsa-miR-340-5p | |  |
|  |  | | hsa-miR-3127-5p | |  |
|  |  | | hsa-miR-6794-5p | |  |
|  |  | | hsa-miR-6076 | |  |
|  |  | | hsa-miR-6839-5p | |  |
|  |  | | hsa-miR-1288-3p | |  |
|  |  | | hsa-miR-219a-5p | |  |
|  |  | | hsa-miR-3156-5p | |  |
|  |  | | hsa-miR-150-3p | |  |
|  |  | | hsa-miR-7152-3p | |  |
|  |  | | hsa-miR-5088-5p | |  |
|  |  | | hsa-miR-6807-5p | |  |
|  |  | | hsa-miR-4701-3p | |  |
| **25µM CLZ Vs control (downregulated)** | | | | | |
| **ABC transporters** | **Drug metabolism cytochrome P450** | | **Drug metabolism other enzymes** | | **Metabolic pathways** |
| hsa-miR-6851-3p | hsa-miR-6851-3p | | hsa-miR-6851-3p | | hsa-miR-6851-3p |
| hsa-miR-339-5p | hsa-miR-1908-3p | | hsa-miR-1908-3p | | hsa-miR-1908-3p |
| hsa-miR-6785-3p | hsa-miR-339-5p | | hsa-miR-339-5p | | hsa-miR-339-5p |
| hsa-miR-6824-3p | hsa-miR-6824-3p | | hsa-miR-6824-3p | | hsa-miR-6824-3p |
| hsa-miR-7114-3p | hsa-miR-6798-3p | | hsa-miR-7114-3p | | hsa-miR-6798-3p |
| hsa-miR-6757-3p | hsa-miR-7114-3p | | hsa-miR-6757-3p | | hsa-miR-7114-3p |
| hsa-miR-6756-3p | hsa-miR-6757-3p | | hsa-miR-6756-3p | | hsa-miR-6757-3p |
| hsa-miR-6819-3p | hsa-miR-6756-3p | | hsa-miR-6819-3p | | hsa-miR-6756-3p |
| hsa-miR-4749-3p | hsa-miR-6819-3p | | hsa-miR-4749-3p | | hsa-miR-6819-3p |
| hsa-miR-4769-3p | hsa-miR-4749-3p | | hsa-miR-4769-3p | | hsa-miR-4749-3p |
| hsa-miR-6763-3p | hsa-miR-4769-3p | | hsa-miR-6763-3p | | hsa-miR-4769-3p |
| hsa-miR-7974 | hsa-miR-6763-3p | | hsa-miR-7974 | | hsa-miR-6763-3p |
| hsa-miR-6812-3p | hsa-miR-7974 | | hsa-miR-1470 | | hsa-miR-7974 |
| hsa-miR-5571-5p | hsa-miR-5571-5p | | hsa-miR-5571-5p | | hsa-miR-1470 |
| hsa-miR-6737-3p | hsa-miR-6737-3p | | hsa-miR-6515-3p | | hsa-miR-6812-3p |
| hsa-miR-6132 | hsa-miR-6132 | | hsa-miR-4313 | | hsa-miR-5571-5p |
| hsa-miR-6515-3p | hsa-miR-6515-3p | | hsa-miR-6069 | | hsa-miR-1281 |
| hsa-miR-4313 | hsa-miR-4313 | | hsa-miR-6858-3p | | hsa-miR-6737-3p |
| hsa-miR-6858-3p | hsa-miR-6069 | | hsa-miR-1275 | | hsa-miR-6132 |
| hsa-miR-1275 | hsa-miR-6858-3p | | hsa-miR-6777-3p | | hsa-miR-6515-3p |
| hsa-miR-6777-3p | hsa-miR-1275 | | hsa-miR-6870-3p | | hsa-miR-4313 |
| hsa-miR-6848-3p | hsa-miR-6777-3p | | hsa-miR-6848-3p | | hsa-miR-6069 |
| hsa-miR-4758-3p | hsa-miR-6870-3p | | hsa-miR-4758-3p | | hsa-miR-6858-3p |
| hsa-miR-1237-3p | hsa-miR-4758-3p | | hsa-miR-6880-3p | | hsa-miR-1275 |
| hsa-miR-3613-3p | hsa-miR-6880-3p | | hsa-miR-4652-3p | | hsa-miR-6777-3p |
| hsa-miR-33b-3p | hsa-miR-1237-3p | | hsa-miR-3613-3p | | hsa-miR-6870-3p |
| hsa-let-7f-1-3p | hsa-miR-4652-3p | | hsa-miR-33b-3p | | hsa-miR-6848-3p |
| hsa-miR-6813-3p | hsa-miR-3613-3p | | hsa-let-7f-1-3p | | hsa-miR-4758-3p |
|  | hsa-miR-33b-3p | | hsa-miR-6813-3p | | hsa-miR-6880-3p |
|  | hsa-let-7f-1-3p | |  | | hsa-miR-1237-3p |
|  | hsa-miR-6813-3p | |  | | hsa-miR-4652-3p |
|  |  | |  | | hsa-miR-3613-3p |
|  |  | |  | | hsa-miR-33b-3p |
|  |  | |  | | hsa-let-7f-1-3p |
|  |  | |  | | hsa-miR-6813-3p |
| **25µM HLP+ 25µM CLZ Vs control (upregulated)** | | | | | |
| **ABC transporters** | **Drug metabolism cytochrome P450** | | **Drug metabolism other enzymes** | | **Metabolic pathways** |
| hsa-miR-629-3p | hsa-miR-4419a | | hsa-miR-629-3p | | hsa-miR-629-3p |
| hsa-miR-4419a | hsa-miR-6759-3p | | hsa-miR-6759-3p | | hsa-miR-4419a |
| hsa-miR-6759-3p | hsa-miR-150-3p | | hsa-miR-3141 | | hsa-miR-6759-3p |
| hsa-miR-150-3p | hsa-miR-3141 | | hsa-miR-4633-5p | | hsa-miR-150-3p |
| hsa-miR-3141 | hsa-miR-4633-5p | | hsa-miR-6743-3p | | hsa-miR-3141 |
| hsa-miR-4633-5p | hsa-miR-6743-3p | | hsa-miR-4270 | | hsa-miR-4633-5p |
| hsa-miR-4270 | hsa-miR-4270 | | hsa-miR-1288-3p | | hsa-miR-6743-3p |
| hsa-miR-1288-3p | hsa-miR-1288-3p | | hsa-miR-5088-5p | | hsa-miR-4270 |
| hsa-miR-5088-5p | hsa-miR-5088-5p | | hsa-miR-7152-3p | | hsa-miR-6834-3p |
| hsa-miR-7152-3p | hsa-miR-7152-3p | | hsa-miR-3156-5p | | hsa-miR-1288-3p |
| hsa-miR-3156-5p | hsa-miR-3156-5p | | hsa-miR-6794-5p | | hsa-miR-5088-5p |
| hsa-miR-6807-5p | hsa-miR-6794-5p | | hsa-miR-6807-5p | | hsa-miR-7152-3p |
|  | hsa-miR-6807-5p | |  | | hsa-miR-3156-5p |
|  |  | |  | | hsa-miR-6794-5p |
|  |  | |  | | hsa-miR-6807-5p |
| **25µM HLP+ 25µM CLZ Vs control (downregulated)** | | | | | |
| **ABC transporters** | **Drug metabolism cytochrome P450** | | **Drug metabolism other enzymes** | | **Metabolic pathways** |
| hsa-miR-339-5p | hsa-miR-339-5p | | hsa-miR-339-5p | | hsa-miR-339-5p |
| hsa-miR-34a-3p | hsa-miR-34a-3p | | hsa-miR-4532 | | hsa-miR-34a-3p |
| hsa-miR-664a-3p | hsa-miR-4532 | | hsa-miR-664a-3p | | hsa-miR-4532 |
| hsa-miR-7974 | hsa-miR-664a-3p | | hsa-miR-7974 | | hsa-miR-664a-3p |
| hsa-miR-10a-5p | hsa-miR-7974 | | hsa-miR-7-1-3p | | hsa-miR-7974 |
| hsa-miR-7-1-3p | hsa-miR-7-1-3p | | hsa-miR-6848-3p | | hsa-miR-10a-5p |
| hsa-miR-6848-3p | hsa-miR-4758-3p | | hsa-miR-4758-3p | | hsa-miR-7-1-3p |
| hsa-miR-4758-3p | hsa-miR-29b-1-5p | | hsa-miR-29b-1-5p | | hsa-miR-6848-3p |
| hsa-miR-29b-1-5p | hsa-miR-371b-5p | | hsa-miR-371b-5p | | hsa-miR-4758-3p |
| hsa-miR-371b-5p |  | |  | | hsa-miR-29b-1-5p |
|  |  | |  | | hsa-miR-371b-5p |
